# Hemoptysis from complex pulmonary aspergilloma treated by cavernostomy and thoracoplasty

**DOI:** 10.1186/s12893-019-0650-1

**Published:** 2019-12-05

**Authors:** Nguyen Truong Giang, Le Tien Dung, Nguyen Thanh Hien, Truong Thanh Thiet, Phan Sy Hiep, Nguyen The Vu, Dinh Cong Pho, Nguyen Van Nam, Pham Ngoc Hung

**Affiliations:** 1 Department of Cardiothoracic Surgery, Military Hospital 103, Vietnam Military Medical University, Hanoi, Vietnam; 2 Department of Thoracic Surgery, Pham Ngoc Thach Hospital, Ho Chi Minh City, Vietnam; 3 Faculty of Medicine, Vietnam Military Medical University, Ha Dong District, Hanoi, Vietnam; 4 Department of Epidemiology, Vietnam Military Medical University, Hanoi, Vietnam; 5 Department of Training, Vietnam Military Medical University, 160 Phung Hung, Ha Dong District, Hanoi, 100000 Vietnam

**Keywords:** Complex pulmonary Aspergilloma (CPA), Hemoptysis, Cavernostomy, Thoracoplasty, Table tennis balls, Tissue expander

## Abstract

**Background:**

In high-risk patients with complex pulmonary aspergilloma but unable for lung resection, cavernostomy and thoracoplasty could be performed. This study aimed to evaluate this surgery compared two compressing materials.

**Methods:**

A total of 63 in high-risk patients who suffered from hemoptysis due to complex pulmonary aspergilloma and underwent cavernostomy and thoracoplasty surgery from November 2011 to September 2018 at Pham Ngoc Thach hospital were evaluated prospectively studied. Patients were allocated to two groups: the table tennis ball group and tissue expander group. We evaluated at the time of before operation, 6 months and 24 months after operation.

**Results:**

Tuberculosis was the most common comorbidity diseases in both groups. Upper lobe occupied almost in location. Hemoptysis symptoms plunged from time to time. Statistically significant Karnofsky score was observed in both groups. Postoperative pulmonary functions (FVC and FEV1) have remained in both groups at all time points. The remarkable results were no deaths related to surgery and low complications both short and long-term. There was no statistical significance between two groups in operative time, blood loss during operation, ICU length-stay time. Four patients died because of co-morbidity in 24 months follow-up.

**Conclusion:**

Cavernostomy and thoracoplasty was safe and effective surgery for the treatment of complex pulmonary aspergilloma with hemoptysis in high-risk patients. No mortality related to surgery and low complications were recorded. The was no inferiority when compared two compressing materials .

## Background

In developing countries, pulmonary aspergilloma is a common disease [1, 2] that difficult to manage because of the low effective in medical treatment [3]. In the challenging clinical situation, surgical treatment emerged as the priority choice that offered good outcomes with acceptable morbidity [4]. Another study showed that it was the most effective treatment [5]. Surgery became a valid indication but choosing the types of surgery depending on many factors.

In low-risk patients, the first choice was pulmonary resection [5], which considered as an appropriate therapy for simple pulmonary aspergilloma with low morbidity and mortality [6–8]. However, it was not the preferred therapy in high-risk patients such as complex pulmonary aspergilloma which has a thick wall of aspergilloma or underlying pleural and parenchymal sequelae. In these patients especially when hemoptysis occurs, immediate treatment is critical because of life-threatening. Anti-fungal therapy and bronchial artery intervention did not show precise results in this situation [9]. Pulmonary resection was considered to perform, but morbidity must be remarked [10]. In the case of unable for lung resection, cavernostomy could be performed [11]. It also was an effective therapy in high-risk patients such as pulmonary function insufficiency, poor general condition, bilateral disease, complex pulmonary aspergilloma [5, 6, 11–13]. Cavernostomy was less invasive procedures with the technically easy, simple and effective procedure, with many advantages [12, 14, 15]. But the cavity that formed after cavernostomy may lead to recurrence. It can be resolved by thoracoplasty using compressing material (also known as plombage surgery). This study presented the details of high-risk patients who underwent cavernostomy and thoracoplasty using compressing material for complex pulmonary aspergilloma with hemoptysis.

## Methods

We evaluated 63 high-risk patients who suffered from hemoptysis due to complex pulmonary aspergilloma (fungal ball) and underwent cavernostomy and thoracoplasty from November 2011 to September 2018. Patients were divided into two groups. Table tennis ball (TTB) group was 46 patients who used table tennis balls, and tissue expander (TE) group was 17 patients who used tissue expander as the compressing material.

Our inclusion criteria were as follows: patients aged above 18 years and had hemoptysis (massive or recurrent) due to complex pulmonary aspergilloma (CPA) and poor general condition, patients with CPA diagnosed on the basis of typical clinical symptoms, conventional X-ray and/or computed tomography images, some tests (bronchoscopy, biochemistry, microbiology) and pathological confirmation after surgery. Cavernostomy and thoracoplasty was recommended on the basis of patients had at least one of following: hemoptysis (massive or recurrent) possibly becoming life-threatening; poor general condition (BMI index <18.5, Karnofsky score < 70), compromised pulmonary function (forced expiratory volume in 1 s [FEV1] < 50.0% or < 1.5 L), surgeon decision depends on the condition of complex pulmonary aspergilloma. Patients who agreed to participate in this study with surgery and follow-up in accordance with the protocol that had been approved by our ethics committee.

Patients who refused to participate and underwent any procedure or surgery concurrent with our surgery were excluded.

We also evaluated*:* patients demographics, clinical and surgical characteristics, postoperative outcomes, and postoperative complications. We followed up the patients at three-time points: before operation, 6 months after operation and 24 months after operation.

### The operative technique

**Fig. 1:**
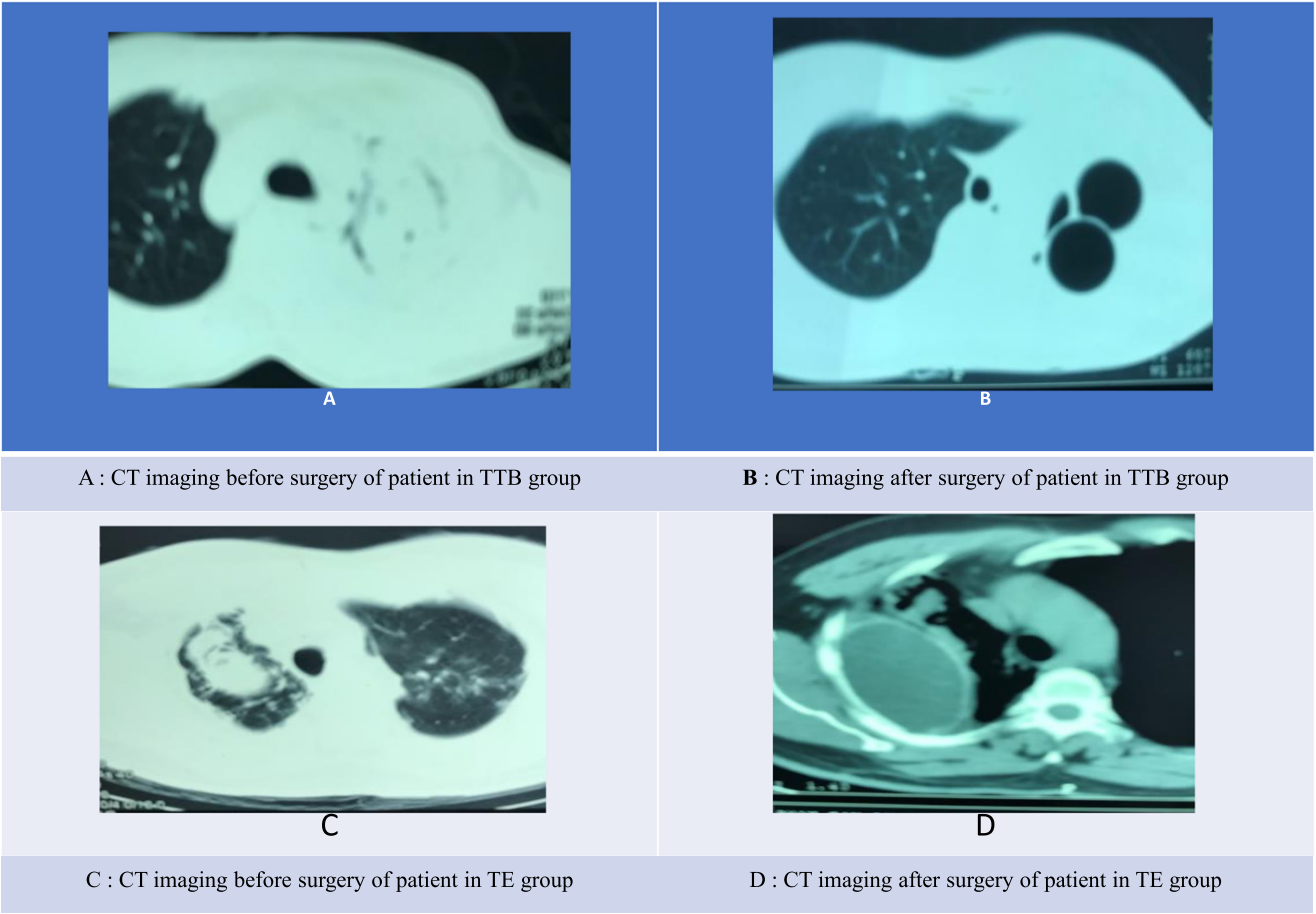
CT imaging before and after surgery

Patients were under general anaesthesia with single-lung ventilation in a lateral decubitus position**.** A metal chest retractor was used to reach complex pulmonary aspergilloma. The fungus ball was removed with a spoon to surgically create a cavity under the ribs. The space was filled by inert material (table tennis ball or tissue expander) to compress the cavern. It also is known as plomgabe surgery or extrapleural pneumonolysis with the principle was that if a diseased lobe of the lungs was physically forced to collapse, then it would heal quickly. In our study, we named table tennis ball or tissue expander as compressing materials. Other necessary techniques were conducted because of lesions. One catheter (24–32 F) was placed into the cavity to control bleeding if necessary. Figure 1 showed CT image before and after operation with two compressing materials to clarify technique.

### The compressing material (Fig. 2: table tennis ball and tissue expander)

We used sterilise table tennis ball (ping-pong) which has been used previously [16] in the TTB group. It is made of a celluloid or plastic material that does not react with human body. It is orange or white and has a diameter of 40 mm, and a weight of 2.7 g [17]. A tissue expander (Polytech Tissue Expander, Polytech Health and Aesthetics GmbH, Germany) is a product approved by the U.S. Food and Drug Administration. We used it in the TE group with the size from 200 to 300 ml and it filled by saline through 23 G needle.

**Fig. 2:**
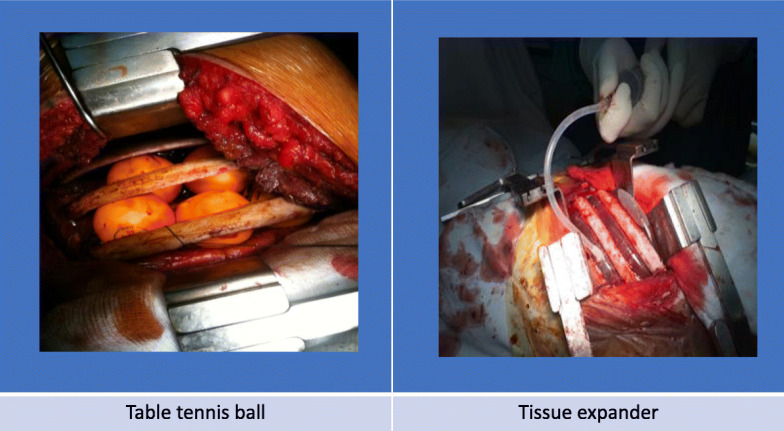
Material used: Table tennis ball (TTB) and Tissue expander (TE)

### Statistical analysis

Data were analyzed with SPSS v21 (IBM Corporation, Armonk, NY, USA). Descriptive analyses were performed with mean and standard deviation. The characteristics were compared between the two groups by using a Student’s *t*-test at a significance level set at 95%. The comparison of serial measurement was performed by two-way ANOVA test.

## Results

A total of 63 patients underwent cavernostomy and thoracoplasty for CPA in Pham Ngoc Thach Hospital, Ho Chi Minh City, Vietnam, from November 2011 to September 2018. Their characteristics of both groups are shown in Table 1. There was no statistically significant in age, size of CPA between TTB group and the TE group. Tuberculosis was the most common comorbidity disease in two groups. The upper lobe (both right and left of the lungs) occupied almost location.

**Table 1:** Demographics

Characteristics	TTB group (*n* = 46)	TE group (*n* = 17)
Age, median (range) (*p*-value = 0.396)	51.06 ± 10.95	53.76 ± 11.28
Sex, n (%)		
Male	34 (73.9)	14 (82.4)
Female	12 (26.1)	3 (17.6)
Underline lung disease, n (%)		
Tuberculosis	41	16
Emphysema	2	0
Lung abscess	2	0
Symptoms and sign, n (%)		
Number of hemoptysis in 24 h		
1	11 (23.9%)	1 (5.9%)
2	9 (19.6%)	12 (70.6%)
3	4 (5.4%)	0 (0%)
> = 4	22 (47.8%)	4 (23.5%)
Blood loss in 24 h		
< 30 ml	7 (15.2%)	3 (17.6)
30–200 ml	23 (50.0%)	11 (64.7)
> 200 ml	16 (34.8%)	3 (17.6)
Cough and sputum	25 (54.3%)	9 (52.9%)
Chest pain	30 (65.2%)	9 (52.9%)
Dyspnea	12 (26.1%)	4 (23.5)
Fever	3 (6.5%)	2 (11.8%)
Location in CT finding		
Right upper lobe	20	10
Right middle lobe	1	0
Right lower lobe	1	0
Left upper lobe	27	7
Left lower lobe	3	0
Multiple locations	6	0
Size of CPA in CT Scan (cm) (*p-*value = 0.6912)	7.93 ± 2.20	8.17 ± 1.87

**Fig. 3:**
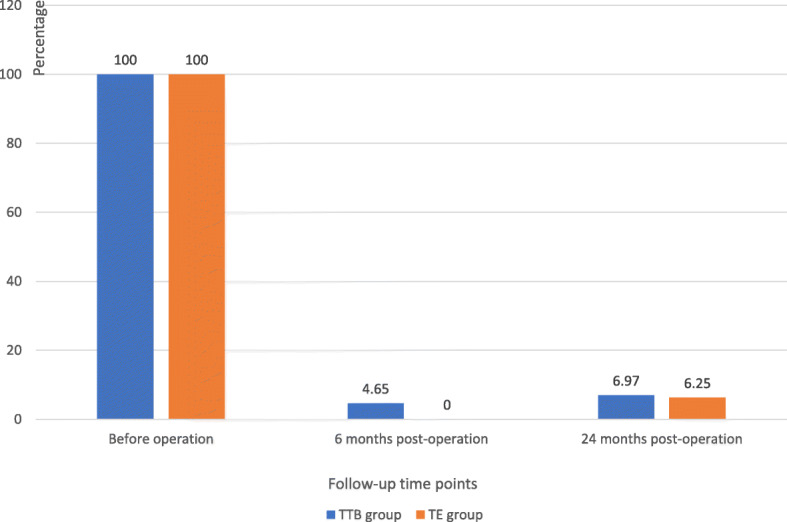
Hemoptysis symptoms before and after operation

**Fig. 4:**
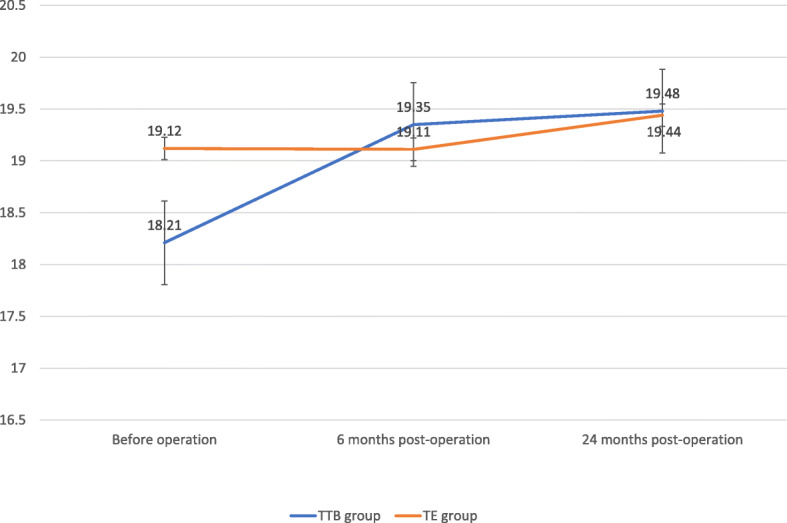
BMI changes before and after operation

**Fig. 5:**
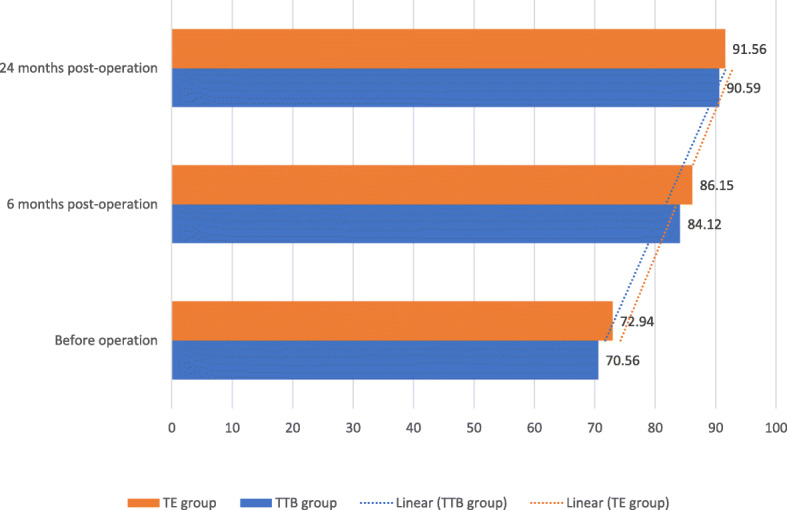
Karnofsky score before and after operation

Hemoptysis symptoms (Fig. 3) plunged from time to time. At 24 months after surgery, hemoptysis ceased 93.03% of patients in TTB group and 93.75% of patients in TE group, diminished 6.97% of patients in TTB group and 6.25% of patients in TE group. Body mass index (Fig. 4) and Karnofsky score (Fig. 5) were compared at the three-time point: before the operation, 6 months after operation, 24 months after operation. No significant differences were shown between the two groups at each time point. While BMI showed slightly increasing but not statistically significant, statistically significant Karnofsky score was observed in both groups. Compared to before operation values, post-operative pulmonary functions (FVC and FEV1) remained in both groups at all time points (Table 2).

**Table 2:** Pulmonary function before and after operation

Index		^(1)^ Before operation	^(2)^ 6 months after operation	^(3)^ 24 months after operation	*p*-value (two times)
FEV1	Group 1 (*n =* 46)	1.43 ± 0.55	1.43 ± 0.52	1.42 ± 0.52	*P*_2–1_ = 1.00 *P*_3–1_ = 0.92
	Group 2 (*n* = 17)	1.36 ± 0.49	1.31 ± 0.42	1.34 ± 0.53	*P*_2–1_ = 0.75 P_3–1_ = 0.90
	*p*-value (two groups)	0.64	0.39	0.59	
FVC	Group 1	2.10 ± 0.63	2.11 ± 0.64	2.07 ± 0.61	*P*_2–1_ = 0.94 *P*_3–1_ = 0.81
	Group 2	2.16 ± 0.566	2.16 ± 0.65	2.13 ± 0.60	*P*_2–1_ = 1.00 *P*_3–1_ = 0.88
	*p*-value (two groups)	0.73	0.78	0.72	

The superscripted numbers "(1, 2 and 3)" related p-value abbreviation. The example is that P2-1: Comparing between before operation and 6 months after operation; P3-1: Comparing between before operation and 24 months after operation.

Surgical characteristics detailed in Table 3. The remarkable results were no deaths related to surgery in the postoperative period, and long-term complication of surgery was low. There was no statistical significance between two groups in operative time, blood loss during operation, ICU length-stay time. The statistically significant only showed in rib retraction.

**Table 3:** Surgical Characteristics

Variables	TTB group (*n* = 46)	TE group (*n* = 17)
Operative time (min) (*p*-value = 0.0796)	135.86 ± 39.47	155.88 ± 39.85
Rib retraction (*p*-value = 0.0296)	3.52 ± 0.65	3.94 ± 0.66
Blood loss during operation (*p*-value = 0.6344)	265.21 ± 193.46	241.17 ± 120.20
Number of table tennis ball (n)	4.34 ± 1.95	
Tissue expander volume (ml)		241.17 ± 53.72
Blood transfusion during operation		
Yes	15	2
No	31	15
Chest drainage		
Yes	54	13
No	21	6
Intra-operative complications		
Pleural tear	1	1
Pneumothorax	0	0
Massive bleeding (≥ 1000 ml)	1	0
Post-operative complications		
Atelectasis	1	0
Pneumothorax	1	0
Tissue expander problems (tear)		1
ICU length-stay time (*p*-value = 0.5606)	2.63 ± 0.95	2.47 ± 1.00
Pathology		
Aspergillus Fumigatus	45	17
Other Aspergillus	1	0
Only aspergilloma	45	0
Other (carcinoma)	1	0

In 63 patients, three patients died in 6 months period after surgery in TTB group because of myocardial infarction, stroke, and lung cancer while in the TE group 1 patient died because of complications of diabetes.

## Discussion

CPA caused many lesions in the lungs that led to life-threatening conditions when complications such as hemoptysis occurred [18, 19]. Immediate treatment was critical for these cases, and surgery was the priority choice if the pulmonary function was not severely insufficiency [20]. Many studies had been carried out to confirm that surgical treatment (almost surgical resection) of pulmonary aspergilloma brought out many advantages such as preventing recurrent hemoptysis and excellent long-term results [2, 3, 8, 21]. Although modern technology using Robotic resection that got further advantages [22] but less invasive surgery such as sub-lobar resection and video-assisted thoracoscopic surgery (VATS) was more preferred with good results just in patients, who had simple pulmonary aspergilloma [23–26].

Anti-fungal medication (voriconazole, itraconazole) was a safe and effective modality and should be considered if surgery contraindicated [27–29]. But in the case of both medical and surgical treatment were ineffective or contraindicated in massive hemoptysis due to aspergilloma, intervention treatment was an alternative therapy with the success rate was 40.0% [30]. Among that, bronchial artery embolism could be considered when systemic embolism was ineffective or to reduce perioperative bleeding [6, 20, 31]. Another therapy such as bronchoscopic procedure and radiotherapy also was a potential option for selected cases [32, 33]. The remarkable result in our study was that the hemoptysis symptoms plunged statistically significant with no cases in the TE group and 2 cases in TTB group. The recurrence rate in 24 months was low, with just only one case in the TE group had hemoptysis, but its severity was lesser than before the operation.

In line with good results of hemoptysis control, Karnofsky score (Karnofsky performance status, Karnofsky Performance Scale) in this study showed statistically significant changes from time point to time point. It was a monitoring index used in peri-operative and post-operative lung transplantation [34, 35]. In our study, the almost pulmonary function of the patient was diminished. After surgery, there was no change in pulmonary function, but the Karnofsky score had significant changes that showed overall efficacy of surgery. The reason behind this may be that hemoptysis was serious sequela in CPA patients that plunged remarkably [20, 36].

In our study, although four patients died, there was no death related to surgery. The reasons for all deaths were a complication of co-morbidity diseases. Another study showed that the mortality rate was variants from one in 17 patients to 4 in nine patients. The reasons behind this may be that the number of patients was small, and the experience of the surgeon may be a lack in this type of surgery [12, 37]. In low-resource countries, surgery for CPA was very challenging, but it was the best treatment modality for symptomatic patients [38]. Lung resection was too invasive and not considered in high-risk patients. When resection was not feasible, alternative therapies, such as cavernostomy [6, 13], intracavitary Amphotericin-B [39] or bronchial artery occlusion [30] should be advised. Cavernostomy was a useful option for high-risk patients with many advantages [11, 20]. The results of our study re-confirmed the efficacy of cavernostomy and thoracoplasty surgery for CPA with hemoptysis.

One of the surgical characteristics was that the cavity formed after carvernostomy and the use of table tennis ball or tissue expander to compress the space and maintain the collapse. This is the difference between our study and other studies using myoplasty. In thoracoplasty, over the years many tissues and materials were tried as a filler, cavernostomy had been performed and showed useful such as single-stage using muscle transposition flap [15, 40], cavernostomy with limited thoracoplasty [15] and simplified cavernostomy involving Alexis Wound Protector [41]. Flap transposition has been approved as component of a multimodal treatment [42] with most used flaps were the latissimus dorsi and the serratus [43]. It permits achieving complete space obliteration [44] for well-selected patients, but in patients with large size cavity or multiple bronchopleural fistulae, it seemed to be ineffective [45]. In our study, the variety of pulmonary size combined with poor general condition seemed to be not suitable for myoplasty. Moreover, the remained space may leaded to recurrence. In our study, we performed single-stage cavernostomy, and thoracoplasty with the recurrence rate was low. This result was in line with Chen et al. [9]. According to our viewpoints, the critical elements of this technique to ensure the recurrence rate was low depended on the bronchial fistula and the cavity condition. The bronchial fistula must be closed that was checked by anesthesia through to expand the lung and no gas leakage if it closed. The cavity was disappeared with the compressed materials (both table tennis ball and tissue expander), losing environment that fungus can be developed.

One key point in our study was compressing materials. Each material has advantages on its own. The table tennis ball emerged as low-cost, easy to find anywhere, but because of the fixed size, it was difficult to manipulate when filled the space. Its complications included shortness of breath, bronchopleural fistula extrusion, superior vena cava obstruction, hemorrhage, pain [16]. There was a report showed that it still works after 46 years with uncomplicated outcome [46]. Tissue expander was used in this surgery as applicable methods. It has been recognized as a standard procedure in the United States for breast reconstruction [47]. Although it has a higher risk of reconstructive failure and surgical-site infection, this was the right choice for high-risk patients or unavailable for autologous reconstruction [48, 49]. This was the reason why we chose tissue expander because of its benefits. We can modify volume to keep fit and ensured that space was compressed appropriately. Several complications of tissue expander in breast reconstruction such as infection, hematoma/seroma, and explantation were reported [50], and there were differences among stages with stage I rather than the later stage [51]. In this study, we performed only one stage with low complications. The results indicate that tissue expander may become a potential material used in cavernostomy and thoracoplasty. The difference between TTB group and TE group was rib retraction. It was statistically significant higher in TE group. The reason behind this was that table tennis ball is fixed and we can add it one by one through appropriate incision but tissue expander is flexible, we need an incision with the same size to put it in the right place.

## Conclusion

Cavernostomy and thoracoplasty was a safe and effective technique for the treatment of complex pulmonary aspergilloma with hemoptysis in high-risk patients. No mortality related to surgery and low complications were recorded. The was no inferiority when compared table tennis ball group and tissue expander group.

## Data Availability

The datasets used and/or analyzed during the current study available from the corresponding author on reasonable request.

## Abbreviations

CPA: Complex pulmonary aspergilloma
TE: Tissue expander
TTB: Table tennis ball
